# Publisher Correction: Across-subject offline decoding of motor imagery from MEG and EEG

**DOI:** 10.1038/s41598-018-30241-y

**Published:** 2018-08-17

**Authors:** Hanna-Leena Halme, Lauri Parkkonen

**Affiliations:** 1 Department of Neuroscience and Biomedical Engineering NBE, Aalto University School of Science, P.O. Box 12200, FI-00076 Aalto, Finland; 2 Aalto Neuroimaging, MEG Core, Aalto University School of Science, Espoo, Finland

Correction to: *Scientific Reports* 10.1038/s41598-018-28295-z, published online 04 July 2018

The original version of this Article contained errors in the spelling of the authors Hanna-Leena Halme and Lauri Parkkonen, which were incorrectly given as Halme Hanna-Leena and Parkkonen Lauri respectively.

These errors have now been corrected in the PDF and HTML versions of the Article.

