# Supplementary material for: Across-subject offline decoding of motor imagery from MEG and EEG
Source: Sci Rep. 2018 Jul 4;8:10087. doi: 10.1038/s41598-018-28295-z (PMC6031658; doi:10.1038/s41598-018-28295-z)
Supplement: Supplementary file 1 — Supplementary information [file 41598_2018_28295_MOESM1_ESM.docx]

**Supplementary information**

**Across-subject offline decoding of motor imagery from MEG and EEG**

Hanna-Leena Halme^a*^, Lauri Parkkonen^a,b^

^a^ Department of Neuroscience and Biomedical Engineering NBE, Aalto University School of Science, P.O. Box 12200, FI-00076 Aalto, Finland

^b^ Aalto Neuroimaging, MEG Core, Aalto University School of Science, Espoo, Finland

***Corresponding author:**

Hanna-Leena Halme

Department of Neuroscience and Biomedical Engineering NBE, Aalto University School of Science, P.O. Box 12200, FI-00076 Aalto, Finland

hanna-leena.halme@aalto.fi

|  | **CSP+LDA** | **CSP+ bagging** | **regCSP** | **Pooling** | **L1-MTL** | **L21-MTL** | **Within- subject** |
| --- | --- | --- | --- | --- | --- | --- | --- |
| **CSP+LDA** | - | 0,16 | **0,02** | **0,02** | **0,04** | **0,03** | **0,00** |
| **CSP+bagging** | 0,16 | - | 0,11 | **0,00** | **0,01** | **0,01** | **0,00** |
| **regCSP** | **0,02** | 0,11 | - | **0,00** | **0,00** | **0,00** | **0,00** |
| **Pooling** | **0,02** | **0,00** | **0,00** | - | 1,00 | 0,51 | 0,07 |
| **L1-MTL** | **0,04** | **0,01** | **0,00** | 1,00 | - | 0,18 | **0,02** |
| **L21-MTL** | **0,03** | **0,01** | **0,00** | 0,51 | 0,18 | - | 0,07 |
| **Within- subject** | **0,00** | **0,00** | **0,00** | 0,07 | **0,02** | 0,07 | - |

Table S1. Statistical differences (*p*-values, calculated by paired t-tests and Bonferroni-corrected for multiple comparisons) between the methods for MI-trained MEG data. Statistically significant differences are in bold.

|  | **CSP+LDA** | **CSP+ bagging** | **regCSP** | **Pooling** | **L1-MTL** | **L21-MTL** | **Within- subject** |
| --- | --- | --- | --- | --- | --- | --- | --- |
| **CSP+LDA** | - | 0,42 | 0,05 | 0,39 | 0,19 | 0,09 | **0,04** |
| **CSP+ bagging** | 0,42 | - | 0,10 | 0,22 | 0,10 | **0,04** | **0,02** |
| **regCSP** | 0,05 | 0,10 | - | **0,04** | **0,01** | **0,01** | **0,00** |
| **Pooling** | 0,39 | 0,22 | **0,04** | - | 0,65 | **0,01** | 0,07 |
| **L1-MTL** | 0,19 | 0,10 | **0,01** | 0,65 | - | 0,15 | 0,10 |
| **L21-MTL** | 0,09 | **0,04** | **0,01** | **0,01** | 0,15 | - | 0,49 |
| **Within- subject** | **0,04** | **0,02** | **0,00** | 0,07 | 0,10 | 0,49 | - |

Table S2. Statistical differences (*p*-values, calculated by paired t-tests and Bonferroni-corrected for multiple comparisons) between the methods using MI-trained EEG data. Statistically significant differences are in bold.

|  | **CSP+LDA** | **CSP+ bagging** | **regCSP** | **Pooling** | **L1-MTL** | **L21-MTL** | **Within- subject** |
| --- | --- | --- | --- | --- | --- | --- | --- |
| **CSP+LDA** | - | 0,86 | 0,62 | 0,74 | 0,12 | 0,17 | **0,01** |
| **CSP+ bagging** | 0,86 | - | 0,77 | 0,82 | 0,25 | 0,28 | **0,03** |
| **regCSP** | 0,62 | 0,77 | - | 0,95 | 0,10 | 0,15 | **0,00** |
| **Pooling** | 0,74 | 0,82 | 0,95 | - | 0,05 | 0,06 | **0,04** |
| **L1-MTL** | 0,12 | 0,25 | 0,10 | 0,05 | - | 0,95 | **0,00** |
| **L21-MTL** | 0,17 | 0,28 | 0,15 | 0,06 | 0,95 | - | **0,00** |
| **Within- subject** | **0,01** | **0,03** | **0,00** | **0,04** | **0,00** | **0,00** | - |

Table S3. Statistical differences between the methods (*p*-values, calculated by paired t-tests and Bonferroni-corrected for multiple comparisons) for PM-trained MEG data. Statistically significant differences are in bold.

|  | **CSP+LDA** | **CSP+ bagging** | **regCSP** | **Pooling** | **L1-MTL** | **L21-MTL** | **Within- subject** |
| --- | --- | --- | --- | --- | --- | --- | --- |
| **CSP+LDA** | - | 0,59 | 0,64 | **0,01** | 0,51 | 0,19 | **0,00** |
| **CSP+ bagging** | 0,59 | - | 0,50 | **0,02** | 0,42 | 0,19 | **0,00** |
| **regCSP** | 0,64 | 0,50 | - | **0,01** | 0,80 | 0,34 | **0,00** |
| **Pooling** | **0,01** | **0,02** | **0,01** | - | **0,04** | 0,22 | **0,00** |
| **L1-MTL** | 0,51 | 0,42 | 0,80 | **0,04** | - | 0,32 | **0,00** |
| **L21-MTL** | 0,19 | 0,19 | 0,34 | 0,22 | 0,32 | - | **0,00** |
| **Within- subject** | **0,00** | **0,00** | **0,00** | **0,00** | **0,00** | **0,00** | - |

Table S4. Statistical differences between the methods (*p*-values, calculated by paired t-tests and Bonferroni-corrected for multiple comparisons) for PM-trained EEG data. Statistically significant differences are in bold.
